# EEG Analysis in Structural Focal Epilepsy Using the Methods of Nonlinear Dynamics (Lyapunov Exponents, Lempel–Ziv Complexity, and Multiscale Entropy)

**DOI:** 10.1155/2020/8407872

**Published:** 2020-02-11

**Authors:** Tatiana V. Yakovleva, Ilya E. Kutepov, Antonina Yu Karas, Nikolai M. Yakovlev, Vitalii V. Dobriyan, Irina V. Papkova, Maxim V. Zhigalov, Olga A. Saltykova, Anton V. Krysko, Tatiana Yu Yaroshenko, Nikolai P. Erofeev, Vadim A. Krysko

**Affiliations:** ^1^Department of Mathematics and Modelling, Yuri Gagarin State Technical University of Saratov, Saratov 410054, Russia; ^2^Medical Center of Neurology, Diagnosis and Treatment of Epilepsy “Epineiro”, Saratov 410054, Russia

## Abstract

This paper analyzes a case with the patient having focal structural epilepsy by processing electroencephalogram (EEG) fragments containing the “sharp wave” pattern of brain activity. EEG signals were recorded using 21 channels. Based on the fact that EEG signals are time series, an approach has been developed for their analysis using nonlinear dynamics tools: calculating the Lyapunov exponent's spectrum, multiscale entropy, and Lempel–Ziv complexity. The calculation of the first Lyapunov exponent is carried out by three methods: Wolf, Rosenstein, and Sano–Sawada, to obtain reliable results. The seven Lyapunov exponent spectra are calculated by the Sano–Sawada method. For the observed patient, studies showed that with medical treatment, his condition did not improve, and as a result, it was recommended to switch from conservative treatment to surgical. The obtained results of the patient's EEG study using the indicated nonlinear dynamics methods are in good agreement with the medical report and MRI data. The approach developed for the analysis of EEG signals by nonlinear dynamics methods can be applied for early detection of structural changes.

## 1. Introduction

Epilepsy is a common neurological disease characterized by sudden various seizures. This disease affects approximately 1% of the world's population. Disease symptoms can begin suddenly, which endanger the life of a person with epilepsy. Early disease diagnosis can improve not only the quality of life, but also save the patient from an accident. When diagnosing a disease, it is important to identify the focus or foci of the disease. Studies are performed on EEG and MRI basis. The EEG signal captures time-varying brain activity impulses. In fact, such signals are chaotic time series. Their randomness can be estimated using methods of nonlinear dynamics that are widely used in other modern science branches, for example, in radiophysics [1], mechanics [2–4], history [5], and others.

One of the characteristics that make it possible to evaluate the chaotic state of a system is the Lyapunov exponent. When examining the signal state, as a rule, either the first exponent or the spectrum of Lyapunov exponents is calculated. A sign of a chaotic state is the positive values of the Lyapunov exponent. Due to this, many authors opt for this approach. Very often, when studying EEG signals, a short-term Lyapunov exponent (STLmax) is used. The authors of [6, 7] use the modified method proposed by the authors to assess the highest short-term Lyapunov exponent STLmax in order to identify signs of a preseizures state. The minimum Lyapunov exponent value indicates well enough the time of the seizure. The Lyapunov exponent is estimated using the Kolmogorov–Smirnov test, which eliminates arbitrary extraneous parameters and gives more stable results. In [8], using the STLmax method [9] has classified the EEG signal, revealing normal or pathological brain activity. Segmentation and calculation of STLmax values is carried out using a trained neural network. Moreover, the overall segments classification accuracy corresponding to normal or pathological activity is 99.6%. In articles [10, 11], the relationship of the STLmax spatial distribution with the onset zone and with the processes leading to the attack is clarified. Based on the results of the calculated STLmax values, topographic modeling is performed. Visual assessment of the STLmax topography helps to determine the location of the onset of the attack. The STLmax values are calculated by the method of [12], in which modification for short-time series was proposed in [13, 14]. In [15], it was revealed that STLmax has the lowest values for the EEG characteristic of a seizure, and the highest values in the post seizure state. In [16, 17], Lyapunov exponents are used to classify the differences between the pre seizure and the post seizure of EEG.

When studying EEG signals, the problem of noise and artifacts arises. For research, the signals that have passed the cleaning are considered as the most suitable. However, this coin has two sides: when cleaning out the signal, important signs can be removed and without them the disease picture will become unreliable. The authors of [18] study EEG signals using the Lyapunov exponent's spectrum and values of the first Lyapunov exponent. They use the method in [12, 19] to determine the first Lyapunov exponent value as a randomness measure of the EEG time series and the Sano–Sawada method [20] to determine the spectrum of Lyapunov exponents to identify signs preceding the seizure. As a result, it was found that the spectrum of Lyapunov exponents is more resistant to signal noise than the first Lyapunov exponent values. In [16], the methodology for training the Elman recurrent neural network (RNN) in combination with Lyapunov exponents is analyzed to classify EEG patterns characteristic of an epileptic seizure and interictal activity. Lyapunov exponents are calculated by the Sano–Sawada method. In [21], to determine pathological changes in EEG signals, an architecture of multilayered perceptron neural network (MLPNN) and exponents was proposed. The work [22] presents a method for modeling the spatiotemporal changes in the brain during epilepsy based on the CML (coupled map lattice) model. The CML optimization method based on the calculation of local and global Lyapunov exponents calculated by the Sano–Sawada method is presented. Based on the results of studying of chaotic EEG signals using the first Lyapunov exponent, the authors of [23, 24] came to the conclusion that the Lyapunov exponent is a reliable chaos measure for low-dimensional or deterministic data (Lorentz system), but when studying data with a high dimension or stochastic nature (EEG signals), it does not give reliable results. In [25], a system for predicting epileptic seizures based on the extraction of correlation dimension, correlation entropy, noise level, Lempel–Ziv complexity, and the highest Lyapunov exponent STLmax for ten patients with focal hippocampal epilepsy was presented. The results showed an average sensitivity of 92.9%.

For the study of EEG signals, the values of the Lempel–Ziv complexity (LZC) are also used. But at present, there is no consensus on the correct interpretation of the results. In [26], the finite dimension of data is studied. The authors provide analytical expressions for the LZC for regular and random sequences and use them to study the effect of finite data size on the LZC. To study the diagnosis of brain pathologies in [27], a comparative study of the complexity of main measures for EEG signals is given. To do this, we study a multiscale complexity measure, depending on the scale, and Lyapunov exponents on the corresponding scales. In [28], a novel complexity measure algorithm, named multiscale permutation Rényi entropy (MPEr), is proposed by introducing the weighting-averaging method.

An analysis of the work shows that there are practically no publications devoted to the study of the patient's EEG signals over a long time interval using nonlinear dynamics methods. Basically, in publications known to us, normal and pathological activity on EEG is studied and EEG patterns characteristic of an epileptic seizure are studied. This work is devoted to the EEG analysis of patients with focal structural epilepsy using nonlinear dynamics methods (Lyapunov and Lempel–Ziv complexity and multiscale entropy) for several years. The authors developed a unified approach to the EEG signal analysis based on the above methods, which can be used for early detection of neurological changes and to identify the patient's whole condition.

## 2. Object of Study

The object of the study is a man born in 1996, who was diagnosed with focal structural epilepsy with focal (cognitive, motor with consciousness impaired) seizures and bilateral tonic-clonic seizures with focal debut, mesial sclerosis on the left, and focal temporal left lobe cortical dysplasia at the age of 8 years. Mother pregnancy and childbirth proceeded without peculiarities, was born on time. Brain injury denied. The epilepsy inheritance is not burdened. Normostenic constitution: height 190 cm; weight 75 kg. Neurological status: psychomotor development corresponds to age, no focal neurological symptoms, funnel chest deformity, and scoliosis of the thoracic spine. The first seizures with impaired consciousness and motor automatisms, periodically with the evolution into bilateral tonic-clonic seizures, appeared from the age of 8. Initially, seizures occurred 1 time per year, usually during a night's sleep, subsequently, the frequency of seizures increased to 3-4 times per month; the last bilateral tonic-clonic seizure was noted at the age of 19 years. The patient complains of frequent seizures with impaired consciousness, which begin with the dizziness appearance, a sudden change in mood, an “influx” of thoughts, and heart palpitations, periodically with subsequent consciousness loss and motor automatisms. The duration of the seizure is up to 30–60 seconds. In the postseizure period, cephalgia, hyperthermia to subfebrile numbers, and drowsiness are noted.

**Figure 1:**
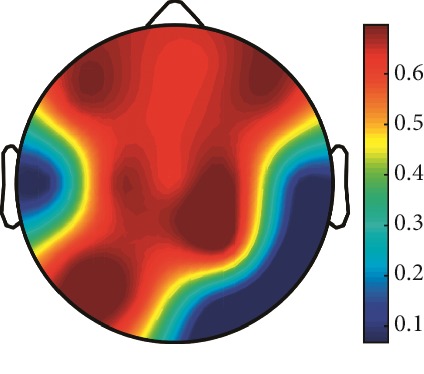
Seizure frequency.

*Therapy*. From childhood, he took long-acting carbamazepine, prolonged-acting valproic acid—without a significant effect—and topiramate, with a positive effect—bilateral tonic-clonic seizures—the number of focal seizures was reduced to 1 per year. From the age of 14, an increase in focal seizures and theirserial flow tendency increased the topiramate dose to 400 mg per day, without a significant effect. In 2014, oxcarbazepine 900 mg/day was added to the treatment, against which there was a significant improvement; over a period of 6 months, a single focal seizure occurred, leading to a gradual reduction in the dose of topiramate that was recommended. In 2015-2016, at a dose of topiramate 200 mg per day in combination with oxcarbazepine 900 mg per day, focal attacks with impaired consciousness became frequent up to 1 time per month and bilateral tonic-clonic seizures resumed, in connection with which it was recommended to increase the daily dose of topiramate. Prescribing topiramate 250 mg per day in combination with the previous dose of oxcarbazepine led to remission of tonic-clonic seizures; however, the focal ones remained with the same frequency throughout 2017. Since 2018, given the increase in focal seizures up to 3-4 times a month, levetiracetam has been added with a gradual increase in dose of 1000 mg per day, which did not bring the expected result, while side effects appeared and began to increase. The cancellation of levetiracetam with an increase in the dose of oxcarbazepine to 1800 mg per day was recommended, which led to a reduction in focal seizures during the first half of 2019 to 1 time in 1.5-2 months (Figure 1).

High-resolution MRI (magnetic-resonance imaging) of the brain according to the epileptological program from 2014 revealed structural changes in the left temporal region in the form of a combination of focal cortical dysplasia of the mediobasal portions of the left temporal lobe and left hippocampal sclerosis, as well as metabolic disorders of the right hippocampus.

**Figure 2:**
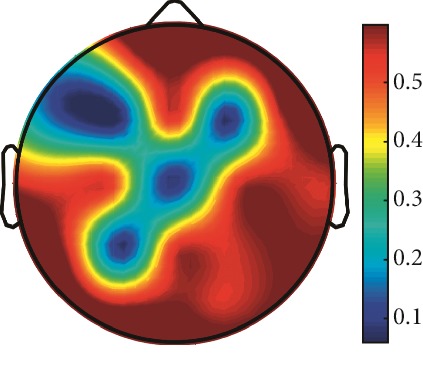
EEG electrode layout on the scalp.

The main diagnostic method, as well as evaluating the effectiveness of treatment for epilepsy, is EEG, in connection with this we will further analyze the patient's EEG signals based on the study of Lyapunov exponent's spectrum and calculation of multiscale entropy and Lempel–Ziv complexity. The EEG of the patient with epilepsy was recorded at the medical center of neurology, diagnosis, and treatment of epilepsy “Epineiro” in Saratov city for 6 years: 2014–2019 (aged 17 to 22 years) on 21 channels: O2, O1, P4, P3, C4, C3, F4, F3, Fp2, Fp1, T6, T5, T4, T3, F8, F7, Pz, Cz, Fz, A2, and A1 with the electrode arrangement shown in Figure 2. Purification from artifacts was carried out by a neurophysiologist. The work analyzes fragments of the patient's EEG containing “sharp wave” complexes since this phenomenon is highly specific for epilepsy. On average, the duration of one signal is 10 seconds and the sampling frequency is 250 Hz.

## 3. Lyapunov Exponent

Lyapunov exponents enable evaluation of the average exponential divergence or convergence of the neighboring trajectories in the phase space. A positive Lyapunov exponent shows that the studied system is chaotic. There is no unified approach for estimation of the Lyapunov exponent; therefore, we used several methods and compared the methods for classic problems [29–31].

### 3.1. First Lyapunov Exponent

Let there be a dynamic system as follows:

(1) $$\begin{array}{c} \dot{x}=f\left(x\right), \end{array}$$

where *x* is an *N*-dimensional state vector.

We choose two close phase points *x*_1_ and *x*_2_ in the phase space, draw trajectories [*x*_1_(*t*)  and  *x*_2_(*t*)], and trace how the distance *d* between the corresponding points of these trajectories changes during the system evolution (1):

(2) $$\begin{array}{c} d\left(t\right)=\left|\overset{⟶}{\varepsilon }\left(t\right)\right|=\left|x_{2}\left(t\right)-x_{1}\left(t\right)\right|. \end{array}$$

If the dynamics of system (1) is chaotic, *d*(*t*) will increase exponentially over time:

(3) $$\begin{array}{c} d\left(t\right)\approx d\left(0\right)e^{kt}. \end{array}$$

From here, we find the average velocity of the trajectories exponential divergence:

(4) $$\begin{array}{c} k\approx \frac{\text{ln }\left[d\left(t\right)/d\left(0\right)\right]}{t}, \end{array}$$

or more precisely

(5) $$\begin{array}{c} k=\underset{\begin{array}{c} d\left(0\right)⟶0\\ t⟶\infty \end{array}}{\lim}\frac{\text{ln}\left[d\left(t\right)/d\left(0\right)\right]}{t}. \end{array}$$

The *h* value (sum of positive exponents) is called the Kolmogorov–Sinai entropy or KS-entropy [32, 33]. Using the KS-entropy, it is possible to determine whether the mode under investigation is chaotic or regular. In particular, if the system dynamics is periodic or quasiperiodic, then the distance *d*(*t*) does not increase with time and KS-entropy is equal to zero (*h* = 0). If the system has a stable fixed point, then *d*(*t*)⟶0 and *h* < 0. In a chaotic system, KS-entropy is greater than zero (*h* > 0).

KS-entropy is a sum of positive Lyapunov's exponents, allowing one to judge the speed information lost about the initial state.

### 3.2. Lyapunov Exponent Spectrum

The spectrum of Lyapunov exponents gives an opportunity to qualitatively assess local stability attractor properties.

Let's take the phase trajectory *x*(*t*) of the dynamical system (1) emerging from the point *x*(0), as well as the trajectory close to it:

(6) $$\begin{array}{c} x_{1}\left(t\right)=x\left(t\right)+\overset{⟶}{\varepsilon }\left(t\right). \end{array}$$

Consider the function

(7) $$\begin{array}{c} \lambda \left[\overset{⟶}{\varepsilon }\left(0\right)\right]=\underset{t⟶\infty }{\lim}\frac{\ln\left[\left|\overset{⟶}{\varepsilon }\left(t\right)\right|/\left|\overset{⟶}{\varepsilon }\left(0\right)\right|\right]}{t}, \end{array}$$

defined on the initial displacement vectors $\overset{⟶}{\varepsilon }\left(0\right)$, such that $\left|\overset{⟶}{\varepsilon }\left(0\right)\right|=\varepsilon$ where *ε*⟶0.

For all possible rotations of the initial displacement vector in *n* directions in the *N*-dimensional phase space, function (7) will change in jumps and take a finite series of values *λ*_1_, *λ*_2_, *λ*_3_,…, *λ*_*n*_. These values of the function *λ* are called Lyapunov exponents. Positive Lyapunov exponents serve as a measure of the average exponential divergence of neighboring trajectories and negative ones as a measure of the average exponential convergence of trajectories to the attractor.

The sum of Lyapunov exponents is the average divergence of the phase trajectories flow, which for a dissipative system (i.e., a system having an attractor) should always be negative. As numerical examples show, for some dissipative systems, Lyapunov exponents are invariant with respect to all enumerated initial conditions. Therefore, Lyapunov exponent spectrum can be considered a property of the attractor.

Usually Lyapunov's exponents are arranged in the descending order. For example, the symbols (+, 0, −) mean that for some attractor in the three-dimensional state space, exponential stretching occurs along one direction, and the phase flow is neutral along the other and undergo exponential compression along the third trajectory direction. It is important to note that attractors other than stable stationary points always have at least one Lyapunov exponent equal to zero since on average, the points on the trajectory are bounded by a compact set and can neither diverge very far nor accumulate.

Consider the relationship of Lyapunov exponents with the properties and types of attractors.

1. *n* = 1. An attractor can only be a stable fixed point (node or focus). In this case, there is one Lyapunov exponent *λ*_1_ = (–) exists.
2. *n* = 2. In two-dimensional systems, there are two types of attractors: stable fixed points and limit cycles. Lyapunov exponents correspond to
3. (*λ*_1_, *λ*_2_) = (−, −) − a stable fixed point;
4. (*λ*_1_, *λ*_2_) = (0, −) − a stable limit cycle (one of the exponents is equal to zero).
5. *n* = 3. In the three-dimensional phase space, there are four types of attractors: stable points, limit cycles, two-dimensional tori, and strange attractors. Lyapunov exponents correspond to
6. (*λ*_1_, *λ*_2_*λ*_3_) = (−, −, −) − a stable fixed point;
7. (*λ*_1_, *λ*_2_*λ*_3_) = (0, −, −) − a stable limit cycle;
8. (*λ*_1_, *λ*_2_*λ*_3_) = (0, 0, −) − a stable two-dimensional torus;
9. (*λ*_1_, *λ*_2_*λ*_3_) = (+, 0, −) − a strange attractor.

The analytical determination of Lyapunov exponents for most problems is not possible because for this it is necessary to know the analytical solution of the differential equations system. However, there are quite reliable algorithms that allow one to find all Lyapunov exponents using numerical methods.

### 3.3. Methods of Analysis of Lyapunov Exponents

#### 3.3.1. Wolf's Method

In [12], Alan Wolf and his coauthors proposed an algorithm that allows one to estimate nonnegative Lyapunov exponents based on time series. In their work, the authors show that Lyapunov exponents are associated with exponentially fast divergence or convergence of neighboring orbits in phase space. Conceptually, the method is based on a previously developed technique that can only be applied to analytically defined model systems. The long-term growth rates of small-volume elements in the attractor are monitored.

The idea of the method is that the method calculates the highest Lyapunov exponent from a sample of a single coordinate and is used when the equations of the system evolution are unknown and all its phase coordinates cannot be measured.

Let there be a time series $x\left(t\right),\;t=\;\bar{1,\;N}$ of one coordinate measurements of the chaotic process, made at equal time intervals. The time delay *τ* is determined by the mutual information method, and the dimension of the embedding space *m* is determined by the method of the nearest false neighbors. As a result of reconstruction, we obtain a point set in the space *R*^*m*^:

(8) $$\begin{array}{c} x_{i}=\left\{x\left(i\right)\text{,}x\left(i-\tau \right),\;\ldots \;,x\left[i-\left(m-1\right)*\tau \right]\right\}=\left[x_{1}\left(i\right)\text{,}x_{2}\left(i\right)\text{,}\ldots \text{,}x_{m}\left(i\right)\right]\frac{1}{2}, \end{array}$$

where $i=\bar{\left[\left(m-1\right)\tau +1\right],\;N\;}$.

Choose a point from sequence (8) and denote it by *x*_0_. Looking through sequence (8), we can find a point $\overset{˜}{x_{0}}$, such that the relation $\tilde{x_{0}}-x_{0}=\varepsilon_{0}<\varepsilon$ holds true, where ε is a fixed quantity much smaller than the reconstructed attractor size. It is also necessary that the points $x_{0}\text{ and }\tilde{x_{0}}$ should be separated in time. After that, the evolution of these points on the reconstructed attractor is monitored until the distance between them exceeds a predetermined value *ε*_max_. We denote the obtained points as $x_{1}\text{ and }\;\tilde{x_{1}}$, the distance between them as *ε*_0_′, and the evolution time interval as *T*_1_.

After this, sequence (8) is considered again and a point $\tilde{x_{1}^{\prime }}$, close to *x*_1_, is found such that $\tilde{x_{1}^{\prime }}-x_{1}=\;\varepsilon_{1}<\varepsilon$. The vectors $\tilde{x_{1}}-x_{1}\text{ and }\tilde{x_{1}^{\prime }}-x_{1}$ should have, if possible, the same direction. Next, the procedure is repeated for the points $x_{1}\text{ and }\tilde{x_{1}^{\prime }}$.

Repeating the described procedure, a large number of times *M*, the first Lyapunov exponent is evaluated as

(9) $$\begin{array}{c} \lambda ≅\frac{\sum_{k=0}^{M-1}\ln\left(\varepsilon_{k}^{\prime }/\varepsilon_{k}\right)}{\sum_{k=1}^{M}T_{k}}. \end{array}$$

#### 3.3.2. Rosenstein's Method

The Rosenstein method [19] is simple to implement and shows a good calculation speed; however, the result of its work is not a numerical value of λ_1_, but a certain function of time:

(10) $$\begin{array}{c} y\left(i,\Delta t\right)=\frac{1}{\Delta t}\langle \ln\;d_{j}\left(i\right)\rangle ,\\ d_{j}\left(i\right)=\underset{x_{j}}{\min}\left\|x_{j}-x_{j}^{\prime }\right\|, \end{array}$$

where *x*_*j*_ is a current point and *x*_*j*_′ is one of its “neighbors.” The algorithm is based on the relationship of *d*_*j*_ and Lyapunov exponents: *d*_*j*_(*i*) ≈ *e*^*λ*_1_(*i*Δ*t*)^. For evaluation, the nearest neighbor of the current point is used. The first Lyapunov exponent is proposed to be calculated as the inclination angle of its most linear section. Finding such a site turns out to be a nontrivial task, and sometimes it is not possible to indicate such a site at all.

#### 3.3.3. Sano–Sawada Method

This method was proposed in [34, 35]. The method essence is reduced to the following algorithm. A phase space sphere having a small radius *ε* is selected. After a number of iterations *m*, some operator *T*^*m*^ transforms this sphere into an ellipsoid with *a*_1_,…  , *a*_*p*_ semiaxes. The sphere will stretch along the axes *a*_1_,…, *a*_*s*_ > *ε*, where *s* is the number of positive Lyapunov exponents, if any. For sufficiently small *ε*, the operator *T*^*m*^ will be close to the sum of the shift operator and the linear operator *A*. The Lyapunov exponents are estimated by averaging the eigenvalues of the operator *A* over the entire attractor. Suppose that there is some vector *ς*_*j*_. We find the set of vectors {*ς*_*ki*_}(*i*=1,…,*N*) that fall into its neighborhood. We obtain a set of vectors *y*_*i*_ ≡ *ς*_*ki*_ − *ς*_*j*_, where *y*_*i*_ ≤ *ε*. After *m* iterations operator, *T*^*m*^ maps vector *ς*_*j*_ into the vector *ς*_*j*+*m*_, and vector *ς*_*ki*_ maps into the vector *ς*_*k*_*i*+*m*__. Consequently, the vectors *y*_*i*_ transform into *y*_*i*+*m*_=*ςk*_*i*+*m*_ − *ς*_*j*+*m*_. If the radius *ε* is small enough, we can assume that there is a linear operator *A*_*j*_ such that *y*_*i*+*m*_=*A*_*j*_*y*_*i*_. Operator *A*_*j*_ describes the system in variations. To estimate the operator *A* we use the least squares method:

(11) $$\begin{array}{c} \underset{A_{j}}{\min}S=\underset{A_{j}}{\min}\frac{1}{N}{\overset{N}{\underset{i=0}{\sum }}\left(y_{i+m}-A_{j}y_{i}\right)}^{2}. \end{array}$$

We obtain an equation system of dimension *n* × *n* of the following form:

(12) $$\begin{array}{c} A_{j}V=C\text{,}{\left(V\right)}_{kl}=\frac{1}{N}\overset{N}{\underset{i=1}{\sum }}y_{i}^{k}y_{i}^{l}\text{,}\\ {\left(C\right)}_{kl}=\frac{1}{N}\overset{N}{\underset{i=1}{\sum }}y_{i+m}^{k}y_{i}^{l}, \end{array}$$

where *V*, *C* are *n* × *n*-dimensional matrices, *y*_*i*_^*k*^ is the *k*-st component of the vector *y*_*i*_, and *y*_*i*+*m*_^*k*^ is the *k*-st component of the vector *y*_*i*+*m*_. Let *A* be a solution to these equations, then Lyapunov exponents can be calculated by the following formula:

(13) $$\begin{array}{c} \lambda_{i}=\underset{n⟶\infty }{\lim}\frac{1}{n\tau }\overset{n}{\underset{j=1}{\sum }}\ln\;A_{j}e_{i}^{j}, \end{array}$$

where {*e*_*j*_} is the set of basis vectors in the tangent space *ς*_*j*_.

When implementing the algorithm, one can do the same as when calculating Lyapunov exponents analytically given systems of ordinary differential equations. An arbitrary basis {*e*^*s*^} is chosen. Next, it is necessary to monitor the change in the vector length *A*_*j*_*e*^*s*^. As the vectors *A*_*j*_*e*^*s*^ grow and their orientation changes, it is necessary to carry out their orthogonalization and renormalization using, for example, the Gram–Schmidt procedure. After receiving a new basis, the procedure is repeated.

### 3.4. Analysis of Classical Systems Using Methods for Calculating Lyapunov Exponents

Since the work is devoted to the study of EEG signals in focal structural epilepsy by analyzing the Lyapunov exponent's spectrum and there is no single developed method for calculating Lyapunov exponents, the question arises of choosing a method that would most accurately allow us to analyze the problem mentioned above. The choice of the method for calculating Lyapunov exponents will be carried out using classic problems as an example: logistic map, Rössler attractor, and Hénon map using three methods: Wolf, Rosenstein, and Sano–Sawada.

#### 3.4.1. Logistic Map

The logistic map [29] describes how the population size changes over time:

(14) $$\begin{array}{c} X_{n+1}=RX_{n}\left(1-X_{n}\right). \end{array}$$

The calculations of the first Lyapunov exponent were carried out for *R* = 4.  Table 1 shows the values of the first Lyapunov exponent. As can be seen from the table, the Rosenstein method and the Sano–Sawada method give the closest results.

#### 3.4.2. Rössler Attractor

The Rössler differential equations are studied [31]:

(15) $$\begin{array}{c} \left\{\begin{array}{c} \dot{x}=-y-z,\\ \dot{y}=x+ay,\\ \dot{z}=b+z\left(x-c\right). \end{array}\right. \end{array}$$

The calculations were performed for the parameters: *a* = *b* = 0.2 and *c* = 5.7.

Table 2 shows the first Lyapunov exponents calculated by three methods, all of them have positive values. Also in Table 2, for the Rössler attractor, the Lyapunov exponent spectrum is calculated by the Sano–Sawada method.

#### 3.4.3. Hénon Map

The Hénon map [30] takes a point with coordinates (*X*_*n*_,  *Y*_*n*_) and maps it to a new point according to the law:

(16) $$\begin{array}{c} X_{n+1}=1-aX_{n}^{2}+\;Y_{n},\\ Y_{n+1}=\;bX_{n}. \end{array}$$

The following parameters were used for the calculation: *a* = 1.4, *b* = 0.3.

Since the equations do not describe any real system, the parameters are simply numbers.  Table 3 shows the first Lyapunov exponents calculated by three methods, all of them have positive values. The Rosenstein method and the Sano–Sawada method give the closest results. Also in Table 3, for Hénon map by the Sano–Sawada method, the spectrum of Lyapunov exponents was calculated.

The values of the highest Lyapunov exponent calculated by the Rosenstein method, the Wolf method, and the Sano–Sawada method for classical problems are in good agreement with each other. Along with the indicated methods for calculating Lyapunov exponents, there is the Kantz method [9] and the modified neural network method [36]. It is worth noting that the calculation of the Lyapunov exponent spectrum using the modification of neural networks takes a longer time compared to the Sano–Sawada method. Due to the fact that the Sano–Sawada method is simpler to implement and takes the least time to calculate, it is most preferable for calculating the spectrum of Lyapunov exponents.

## 4. Multiscale Entropy (MSE)

According to the method of calculating of multiscale entropy (MSE) presented in [37], for a given discrete time series {*x*_1_,…,*x*_*i*_,…,*x*_*N*_}, a sequence from the simplified time series {*y*^(*τ*)^} is determined relative to the scaling parameter *τ*. The initial time series is divided into nonoverlapping windows of length *τ*, and then the values are averaged for each window. Thus, each element of the simplified time series is calculated by the following formula:

(17) $$\begin{array}{c} y_{j}^{\left(\tau \right)}=\frac{1}{\tau }\overset{jr}{\underset{i={\left(j-1\right)}_{\tau }+1}{\sum }}x_{i}\text{,}\;1\leq j\leq \frac{N}{\tau }\text{.} \end{array}$$

For the first scale, the time series {*y*^(1)^} is equivalent to the original time series. The length of each time series corresponds to the length of the original time series divided by the scaling parameter *τ*.

The quantitative measure calculation of entropy *S*_*E*_ for each simplified time series is carried out according to the following formula:

(18) $$\begin{array}{c} S_{E}\left(m,\;r,\;N\right)=\ln\frac{\sum_{i=1}^{N-m}n_{i}^{\prime m}}{\sum_{i=1}^{N-m}n_{i}^{\prime m+1}}, \end{array}$$

where *m* is the increment of the data vector length, *r* is the cell size in the phase space (error), and *n*_*i*_^′*m*^ is the probability of repeating a given length data sequence in the source data.

## 5. Lempel–Ziv Complexity (LZC)

In [38], Lempel and Ziv proposed a measure of the patterns complexity for finite length sequences. Later, Kaspar and Schuster developed an algorithm for computing LZC on a computer, which determined the measure of complexity [39]. LZC calculates the quantity of new images, i.e., segments that are not consistently represented in all previous data. In this algorithm, the EEG signal {*x*(*n*)} is converted into a binary sequence {*s*(*n*)} by comparison with the average value of the signal *m*. After obtaining the binary sequence, the corresponding measure of complexity *c*(*n*) is increased by one until a new sequence is detected. The sequence search process is repeated until the last time the series character has been read. LZC is defined as

(19) $$\begin{array}{c} \text{LZC}=\frac{c\left(n\right)}{b\left(n\right)}\text{,} \end{array}$$

where *b*(*n*)=*n*/log_2_(*n*).

## 6. Analysis of the EEG Signals of a Patient with Epilepsy Using Lyapunov Exponents, Multiscale Entropy, and Lempel–Ziv Complexity

We study fragments of EEG signals containing pathological changes in the “sharp wave” (according to the international classification of EEG disorders, Luders H, Noachtar S, 2000) of the patient, who suffered from epilepsy during 2014–2019 in order to study the general patient condition. EEG recordings were taken once a year in interictal periods. During the recording of the EEG, many factors had been influencing the patient's condition, including changes in therapy: a combination of medicaments and their doses. The study was carried out using nonlinear dynamics methods, namely, the Lyapunov exponent's analysis, multiscale entropy, and Lempel–Ziv complexity.

**Figure 3:**
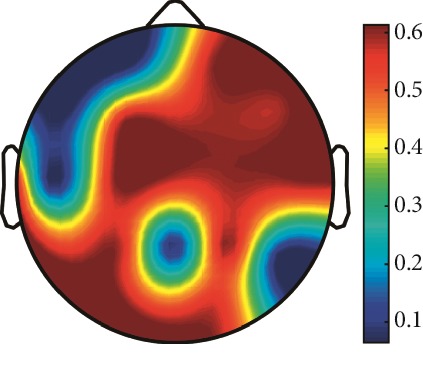
First Lyapunov exponent for 2015.

**Figure 4:**
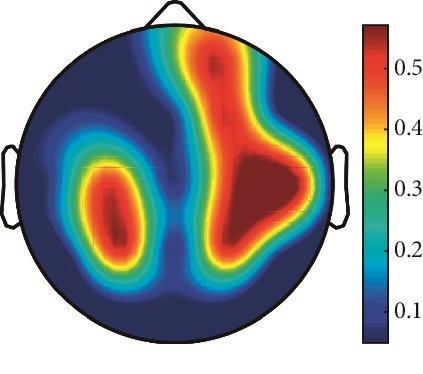
First Lyapunov exponent for 2018.

**Figure 5:**
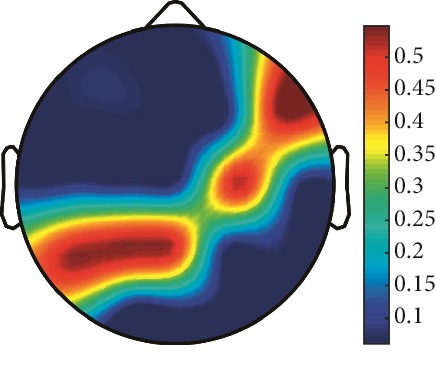
EEG fragment of a patient with epilepsy.

For each year under consideration, Lyapunov's first exponents (Le1) were calculated in each channel by three methods: Rosenstein (blue line), Sano–Sawada (red line), and Wolf (green line). Figures 3 and 4 show the results for 2015 and 2018 years, respectively. The first Lyapunov exponents calculated by the Wolf and Sano–Sawada method are close to each other, and only in some channels the values by the Sano–Sawada and Rosenstein methods coincide. The “sharp wave” pattern on the EEG is observed in the F7 channel (Figure 5), and on the charts of the first Lyapunov exponent calculated by the Rosenstein and Sano–Sawada method, a local minimum is reached in the F7 channel (Figures 3(a) and 4(a)). The Rosenstein method gives the widest range of values across channels [5^∗^ 10^−2^; 6^∗^ 10^−1^] (Figures 3(a) and 4(a)).

In the graph of the Lyapunov exponent calculated by the Rosenstein method, in 2015 (Figure 3(a)), local maxima are observed in the channels P4, C4, C3, Fp2, T6, F8, Pz, and Fz; and in 2018 (Figure 4(a)), only in the channels P3, C4, T5, F8, and Pz, thus the average value becomes closer to zero, which characterizes the disease spread to other brain areas. Qualitatively, the distribution of the first Lyapunov exponent over the channels, calculated by the three methods, coincides. The value of the first Lyapunov exponent calculated by the Sano–Sawada method has a negative value in some channels (Figures 3(a) and 4(a)), which is a measure of the average exponential convergence of the trajectories to the attractor. All this indicates a trend of worsened patient condition. On the joint figures (Figures 3(a) and 4(a)), due to the smallness of the first Lyapunov exponent values calculated by the Wolf method, the line corresponding to it is straightened. Therefore, to see the distribution nature over the channels, a graph of the first Lyapunov exponent is calculated by the Wolf method only (Figures 3(b) and 4(b)). The Wolf method has low sensitivity, and the first Lyapunov exponent values are in the range [−1^∗^ 10^−3^; 3^∗^ 10^−3^], that is, very close to zero. In 2018, according to the Wolf method, the first Lyapunov exponent in channel T3 is zero (Figure 4(b)), i.e., the signal is harmonic, and this fact is in good agreement with the neuroimaging data that the patient has structural changes in the left temporal region.

However, it is worth noting that pathological electrical activity from one brain zone extends to the remaining zones and has a significant effect, so it makes sense to study the average value of the Lyapunov exponent for all channels. In 2014, a “sharp wave” pattern was observed for the patient on the EEG in the F7, T3, and F3 channels, and the response of this wave is also extended to the right hemisphere channels T4, T6, and O2, which is confirmed by the closeness to the zero value of the first Lyapunov exponent in these channels calculated by the Rosenstein method (Table 4). Similar results were obtained for the remaining years, and there is a tendency to increase zones in which the Lyapunov exponent is close to zero, which characterizes the general deterioration of the patient's condition. Table 4 shows the distribution of Lyapunov exponents for each channel over the scalp surface. To visualize the obtained data in the Matlab software package, algorithms for constructing topographic images of Lyapunov exponents were implemented in accordance with electrodes arrangement in Figure 2. The values at the intermediate points were interpolated using a spherical spline. The minimum values are shown in blue and the maximum values in dark red.

**Figure 6:**
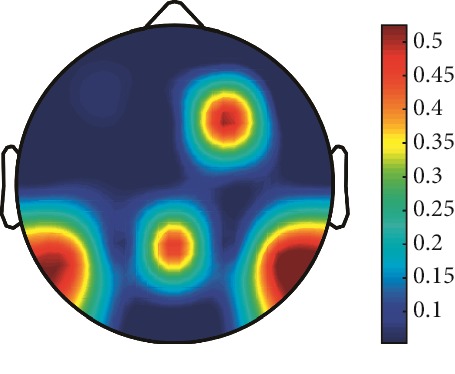
Distribution of the channel-averaged values of the first Lyapunov's exponents by three methods for 2014–2019 years.

Consider the average across all channels of the first Lyapunov exponent calculated by three methods: Rosenstein (blue line), Sano–Sawada (red line), and Wolf (green line). Let us construct the distribution diagrams over the years of the obtained average value for the first Lyapunov exponent (Figure 6). Qualitatively, the Sano–Sawada and Rosenstein methods give similar results (Figure 6(a)). In 2015 and 2017, there are local minima (Figure 6(a)), which characterizes the deterioration of the patient's condition. During this period, the patient had more frequent focal seizures with impaired consciousness and bilateral tonic-clonic seizures resumed (Figure 1). All three methods give positive values of the first Lyapunov exponent; however, the general trend in the values distribution from 2014 to 2019 years indicates the values tend to zero (Figure 6(a)) and the Wolf method gives results very close to zero throughout all years (Figure 6(b)), which indicates a trend towards signals “harmonization”, i.e., worsening of the patient whole condition.

**Figure 7:**
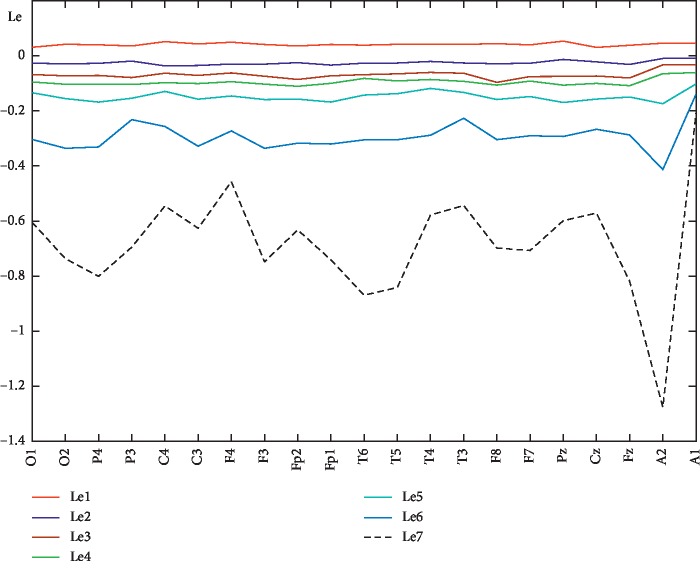
The Lyapunov exponent's spectrum according to the Sano–Sawada method for 2015.

**Figure 8:**
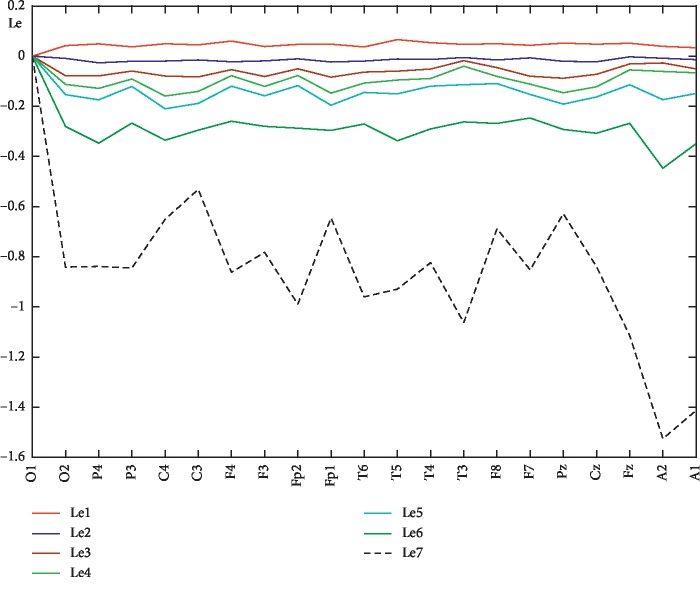
The Lyapunov exponent's spectrum by the Sano–Sawada method in 2018.

**Figure 9:**
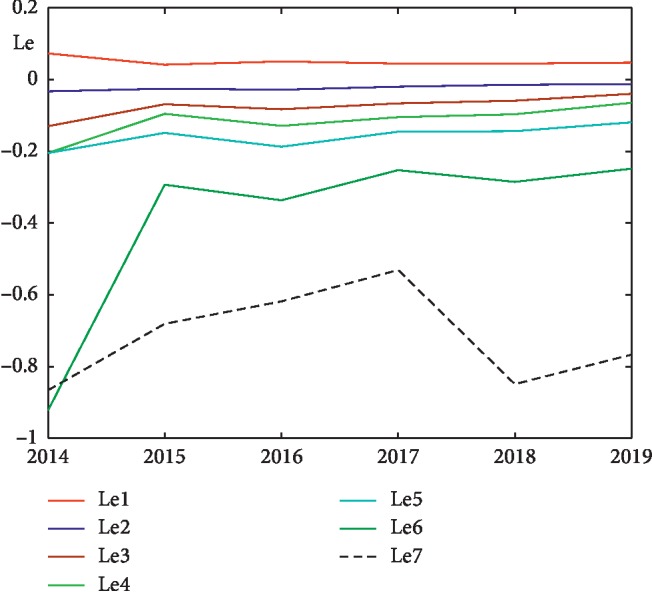
Distribution of the channel-averaged Lyapunov exponents spectrum according to the Sano–Sawada method for the 2014–2019 years.

In the future, due to the fact that all three methods qualitatively show one trend, we will calculate the Lyapunov exponent spectrum by the Sano–Sawada method. It is worth noting that the value of the first Lyapunov exponent depends on the amount of calculated exponents in the spectrum. Here are the graphs of the Lyapunov exponents spectrum (Le1-Le7) calculated by the Sano–Sawada method in each channel for 2015 (Figure 7) and 2018 (Figure 8) years, as well as the spectrum distribution of the averaged Lyapunov exponents over the channels for the years 2014–2019 (Figure 9). As can be seen from the graphs, the first Lyapunov exponent (Le1) has a positive, but close to zero value. The remaining exponents, starting from the second, (Le2-Le7) have negative values. The channel-averaged values of the Lyapunov exponent's spectrum during 2014–2019 (Figure 9) tend to zero, which again indicates a deterioration in the patient's condition.

**Figure 10:**
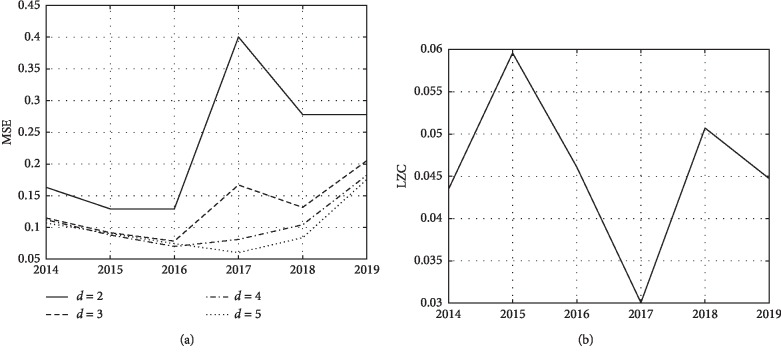
Multiscale entropy (MSE) (a) and Lempel–Ziv complexity (LZC) (b) for the years 2014–2019.

To estimate the EEG signals complexity, we apply algorithms for calculating multiscale entropy (MSE) and the Lempel–Ziv complexity measure (LZC). EEG signals can be represented at various spatial and temporal scales, so their complexity is also multiscale. Given this statement, MSE analysis was carried out for various scales *d*=2,   …,  5 (see Figure 10(a)). The analysis of the plots of the channel-averaged MSE value for every considered year (see Figure 10(a)) shows that the increase of the scaling coefficient above *d*=4 is redundant, as convergence of entropy values is reached. Thus, the following parameters are used for the entropy analysis: embedding dimension *m* = 20, error *r* = 0.4, and scaling coefficient *d* = 3.

When calculating the Lempel–Ziv complexity (LZC), normalization of values was used. A decrease in entropy and an increase in the Lempel–Ziv complexity characterize a decrease in the signal randomness. The average value graph across all channels of the Lempel–Ziv complexity has a local maximum in 2015 and 2018 years (Figure 10(b)), and multiscale entropy has a local minimum in 2016 and 2018 (Figure 10(a)), which is well comparable with the dynamics of patient seizures (Figure 1). In 2017, the patient experienced remission of tonic-clonic seizures, which is in good agreement with the LZC graph (local minimum in 2017) and the MSE graph (local maximum in 2017).

## 7. Conclusion

In the present work, for the first time, an investigation was made for EEG signals containing pathological changes in the “sharp wave” for a patient with focal epilepsy for several years using methods of nonlinear dynamics (analysis of the spectrum of Lyapunov exponents, Lempel–Ziv complexity, and multiscale entropy). A unified approach to the analysis of EEG signals based on the above methods was developed, which can be used for the early detection of structural changes in the brain and treatment effectiveness prediction.

For the first time, to obtain the results reliability, the analysis of Lyapunov exponents was carried out by several different methods (Wolf, Rosenstein, and Sano–Sawada). It was revealed that the Rosenstein method is the most informative for the zones localization of abnormal activity, and the Sano–Sawada method well describes the general trend in the condition change of a patient with epilepsy. Such characteristics as MSE and LZC are informative when comparing the patient's condition in the current year with the previous one.

Using the methods of nonlinear dynamics, it was revealed that the deviations are located in the temporal parts of the hemispheres, which advise the channels T3 and T4. This is confirmed by high-resolution brain MRI according to the epileptological program: structural changes in the temporal region were revealed in the form of a combination of focal cortical dysplasia of the left temporal lobe mediobasal parts and left hippocampal sclerosis, as well as metabolic disorders of the right hippocampus.

The results obtained using the proposed methodology (analysis of the Lyapunov exponents spectrum, multiscale entropy, and Lempel–Ziv complexity) showed deterioration in the patient's condition during the period under review, which is in good agreement with the medical report.

The developed software package based on the proposed methodology can be used in the medical epilepsy diagnosis, as well as a qualitative therapy selection.

## Figures and Tables

**Table 1:** The first Lyapunov exponent for logistic mapping.

The first Lyapunov exponent		
Wolf	Rosenstein	Sano–Sawada
LLE: 0.99683	LLE: 0.690553	LES: 0.69317

**Table 2:** The first Lyapunov exponent for the Rössler attractor.

The first Lyapunov exponent		
Wolf	Rosenstein	Sano–sawada
LLE: 0.05855	LLE: 0.0726	LES: 0.099851; −0.014317; −0.72266

**Table 3:** The first Lyapunov exponent for the Hénon map.

The first Lyapunov exponent		
Wolf	Rosenstein	Jacobian
LLE: 0.38788	LLE: 0.414218	LES: 0.42703; −1.5717

**Table 4:** The first Lyapunov exponent in channels calculated by the Rosenstein.

2014	2015	2016	2017	2018	2019
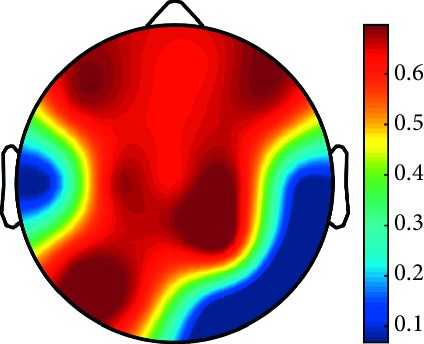	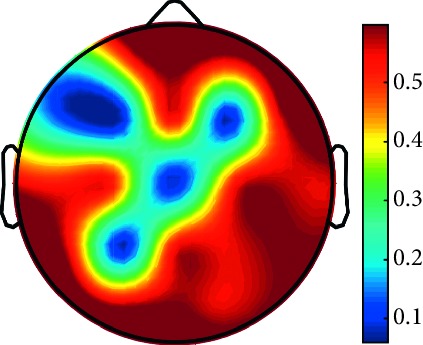	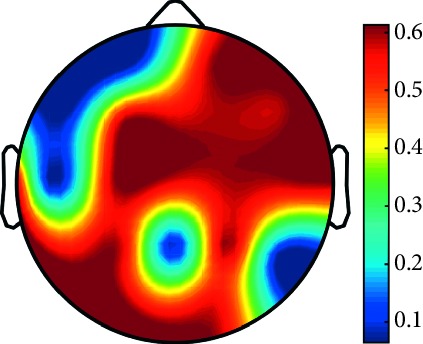	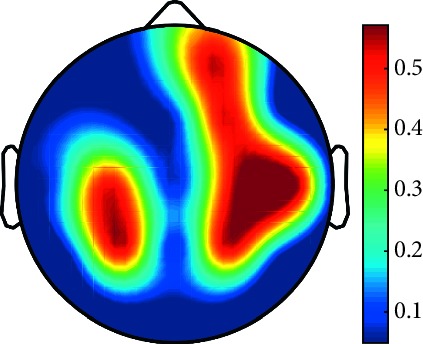	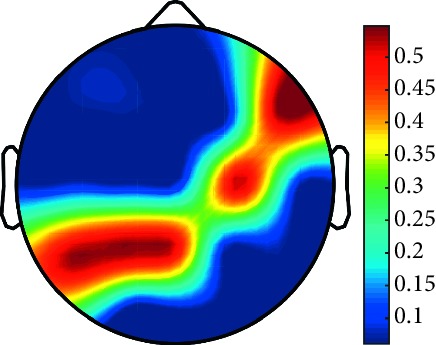	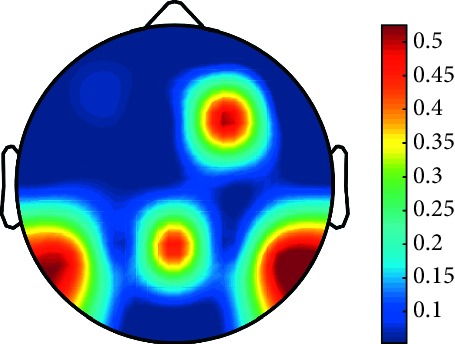

## Data Availability

The data used to support the findings of this study are available from the corresponding author upon request.
